
JOURNAL INFORMATION
==============================
NLM Title Abbreviation: ScientificWorldJournal
Journal ID: ScientificWorldJournal
NLM Journal ID: 101131163
Journal ID: TSWJ

The Scientific World Journal

ISSN: 2356-6140
EISSN: 1537-744X
Publisher: Wiley

ARTICLE INFORMATION
==============================
PMCID: PMC6009689
PMID: 29973822
DOI: 10.1100/tsw.2002.48
Article ID: 439131
Article version: 1
Subjects: Short Report

A Missing Step in Glycogen Synthesis

Park Mari Dei
Debayle Melissa
Whelan William J.
Barquist Erik
Klein Yoram
Departments of Biochemistry and Molecular Biology, and Surgery, University of Miami School of Medicine, P.O. Box 016129 (M823), Miami, FL 33101, USA
Publication date: 2002
Electronic publication date: 2002 Mar 16
Volume: 2
First page: 102
Last page: 103
Copyright: Copyright © 2002 Mari Dei Park et al.
Copyright year: 2002
License: This is an open access article distributed under the Creative Commons Attribution License, which permits unrestricted use, distribution, and reproduction in any medium, provided the original work is properly cited.
License URL: https://creativecommons.org/licenses/by/3.0/

==============================
Advances in Gene Technology: The Genome and Beyond –
Structural Biology for Medicine (Proceedings of the 2002 Miami
*Nature Biotechnology* Winter Symposium)
*TheScientificWorld* 2002, 2(S2), 102–103
ISSN 1532-2246; DOI 10.1100/tsw.2002.48

### A MISSING STEP IN GLYCOGEN SYNTHESIS

Mari Dei Park, Melissa Debayle, William J. Whelan, Erik Barquist, and Yoram Klein
Departments of Biochemistry and Molecular Biology, and Surgery, University of Miami School of Medicine, P.O. Box 016129 (M823), Miami, FL 33101

**INTRODUCTION.** There is strong evidence for the existence of a discrete intermediate in glycogen (macroglycogen) synthesis. This has been termed proglycogen[1]. It is narrowly disperse and has a mass of about 400kDa. Containing 10% by weight of the glycogen primer, the self-glucosylating glycogenin, proglycogen is separable from macroglycogen by the insolubility of the former in 10% trichloracetic acid (TCA). The synthesis of glycogen from glucose in cultured astrocytes can be arrested at proglycogen, as can the degradation of macroglycogen in cultured quail embryo muscle. This suggests the possibility of distinct enzyme species operating between glucose and proglycogen that are different from the classical glycogen synthase and phosphorylase, which until now have been considered the only enzymes involved in extending and pruning the chains of glycogen. The putative proglycogen synthase differs from the already known glycogen synthase by having a much greater affinity for UDPglucose. Rather than postulating that proglycogen synthase is a different gene product, it has been suggested that changes in the pattern of multisite phosphorylation of glycogen synthase might modulate its affinity for UDPglucose[1].

Jiao et al.[2] have reported the presence in human muscle of a form of glycogen synthase that has the same high affinity for UDPglucose as we have reported for proglycogen synthase. Like proglycogen synthase, it incorporates glucose into material precipitated by 10% TCA. This corresponds to glycogenin undergoing glucosylation and conversion into proglycogen. The new synthase is characterized by not being activated by glucose 6-phosphate, which does activate astrocyte proglycogen synthase. Instead, it is activated by manganese sulfate. Both cation and anion are necessary. Thus $MnCl_2$ had little activating effect. We have examined the report by Jiao et al.[2] in terms of whether this new activity could be that of a proglycogen synthase.

**MATERIALS AND METHODS.** Frozen rabbit muscle was obtained from PelFreez. Human cremaster muscle samples were surgically removed from volunteers undergoing treatment for hernia. They were kept under liquid $N_2$ and used immediately or after storage at –80$^o$ C. Labeling of TCA-precipitable material when muscle extracts were incubated with UDP[$^{14}$C]glucose was carried out as by Lomako et al.[3].

**RESULTS.** No glucose-transferring activity could be detected in rabbit muscle that was activated by $MnSO_4$, over and above other Mn salts, e.g. $MnCl_2$ (which activates glycogenin). Accordingly we resorted to human muscle. (Jiao et al.[2] do not mention a reason for using human muscle.)

With cremaster muscle extracts we found evidence of preferential labeling in presence of $MnSO_4$ versus $MnCl_2$, although the relative degrees of labeling (2.1:1) were not as different as reported by Jiao et al.[2], where labeling by $MnCl_2$ was said to be <5% of that in presence of $MnSO_4$. Preferential activation by $MnSO_4$ was also seen when the degree of labeling at various $Mn^{2+}$ concentrations was measured. Optimum labeling at 3mM $MnSO_4$ was noted, as also by Jiao et al.[2].

However, an important discrepancy was noted when muscle extracts were filtered through Sephadex G-25 before being incubated with UDP[$^{14}$C]glucose. Filtration virtually abolished the ability of the extract to glucosylate TCA-precipitable material in presence of $MnSO_4$. The substantial activation of labeling in presence of glucose 6-phosphate was unchanged by filtration.

**DISCUSSION.** The TCA-precipitable material that undergoes $MnSO_4$-activated glucosylation cannot be proglycogen. This is because its removal on Sephadex G-25 demonstrates it to have low molecular weight. That proglycogen, and proglycogen synthase, similar to the astrocyte enzyme, were present in the filtered extracts was shown by the glucose 6-phosphate-activated labeling and by SDS-PAGE and radioautography**.**

A possible candidate for the $MnSO_4$-activated labeling reported by Jiao et al.[2] is an endogenous lipid, also precipitable by TCA. The phenomenon they reported seems unlikely to be connected with glycogen synthesis. The question whether proglycogen synthase is a gene product distinct from macroglycogen synthase remains unanswered.

We thank Dr. J. Lomako for suggesting the filtration experiment.

**REFERENCES**

1. Alonso, M.D., Lomako, J., Lomako, W.M., and Whelan, W.J. (1995) FASEB J. 9, 1126–1127.
2. Jiao, Y., Shashkina, E., Shashkin, P., Hansson, A., and Katz, A. (1999) Biochim. Biophys. Acta 1427, 1–12.
3. Lomako, J., Lomako, W.M., and Whelan, W.J. (1988) FASEB J. 2, 3097–3103.
